# Supplementary material for: The effect of a family-centered advance care planning intervention for persons with cognitive impairment and their family caregivers on end-of-life care discussions and decisions
Source: PLoS One. 2022 Sep 6;17(9):e0274096. doi: 10.1371/journal.pone.0274096 (PMC9447906; doi:10.1371/journal.pone.0274096)
Supplement: Attachment — Submitted filename: Response to Reviewers.docx [file pone.0274096.s002.docx]

July 31, 2021

Helen Chan PhD
Academic Editor

*PlosOne*

**Manuscript ID** PONE-D-21-14530

**Manuscript Title**: *The effect of a family-centered advance care planning intervention for persons with cognitive impairment and their family caregivers on end-of-life care discussions and decisions*

Dear Professor,

The authors appreciate the opportunity to revise our manuscript entitled **“**The effect of a family-centered advance care planning intervention for persons with cognitive impairment and their family caregivers on end-of-life care discussions and decisions**”.** We have made extensive revisions to the manuscript thanks to the valuable comments and suggestions from both reviewers.

To facilitate review, all suggested changes, as well as additional revisions to the manuscript, are in red font. We hope our manuscript is now acceptable for publication in *PLOS ONE*. Our responses are as follows:

**Responses to the comments from Reviewer 1**

Comment 1*: It’s my pleasure to review your manuscript. This paper presents a very important research topic—using a family-centered approach to promote ACP to persons with cognitive impairment (PWCIs) and their family caregivers (FCGs) which has drawn much attention in the research world and clinical practice. The aim of this paper was to evaluate the effectiveness of the ACP intervention on four outcomes: i) Familiarity with ACP; ii) ACP engagement intention; iii) participation in EOL care discussion; iv) dyadic concordance on EOL care decisions between the PWCIs and FCGs.*
**Response 1**: Thank you for your valuable comments. We have followed your suggestions to for the revision of our manuscript.

Comment 2: **Methodology section**
*This section was clear in general. Description of study design and intervention were clear. The eligibility criteria was somewhat clear.*

Comment 2a: *Mind the way of using “or” to link up different conditions. It was somehow misleading that individuals with MMSE≥18 was eligible to participate. Also, in the result section, it was mentioned that two PWCIs had signed AD at baseline. However, according to the eligibility criteria, individuals who have participated in ACP seemed to be excluded from this study.*

**Response 2a**: Thank you for your comment, which drew attention to an error in the methods. We have deleted “or a score ≥ 18 on the Mini-Mental State Examination (MMSE)”. Although PWCIs who had participated in an ACP consultation were excluded from our study, having signed an AD of any kind was not one of the criteria for exclusion. Please see **Response 2b**.

Comment 2b: *Would it be the case in Taiwan if an individual can sign AD without went through ACP? If not, screening of subjects may have some problems.*

**Response 2b:** Thank you for this question. At the suggestion of Reviewer 2, we added two new paragraphs to the introduction to expand the description of the difference between advance care planning and an advance directives (Pages 3-4, lines 52-73):

Dementia is a progressive neurodegenerative disease that is life-limiting. An inevitable consequence of dementia is that the mental status of persons with cognitive impairment (PWCIs) will gradually deteriorate to the end-of-life (EoL) stage [1, 2]. Advanced dementia puts individuals at risk of inadequate EoL-care due to decreases in cognitive and decision-making abilities. Advance care planning (ACP) is one means of improving quality of EoL-care for PWCIs and their family caregivers (FCGs) [1, 3]. ACP is an umbrella term that includes personal, clinical, legal, and financial planning in preparation for the time an individual no longer has the capacity to make their own decisions about healthcare or legal proceedings. The process of ACP often includes advance directives (ADs), which are formal written documents describing preferences for future healthcare, including directives about do-not-resuscitate (DNR), intubation and artificial respiration, and tube feeding, as well as appointing a surrogate to make healthcare decisions in the event of incapacity [1, 2]. However, an individual can provide ADs without ACP, which is more extensive in terms of the number of personal preferences included.

ACP can be conducted formally through consultations with healthcare or legal professionals, or through informal discussions with family members [4]. A recent consensus paper defined ACP for health care as, ‘*the ability to enable individuals to define goals and preferences for future medical treatment and care, to discuss these goals and preferences with family and healthcare providers, and to record and review these preferences if appropriate’* [5]. National experts recommend that formal ACP should address the individual’s concerns and values across the physical, psychological, social, and spiritual domains and to tailor holistic care that responds to the wishes of the individual [5].

Second, the current policy in Taiwan is that both the Hospice Palliative Care Act (HPCA) and Patient Right to Autonomy Act (PRAA) coexist in clinical practice. However, these two Acts differ in the scope of the application and the options for end-of-life care. Indeed, HPCA does not require an ACP consultation before signing an AD. In contrast, the PRAA stipulates that an individual must participate in an ACP consultation before signing AD documents. We have revised the Introduction to clarify the difference (P5, lines 91-101):

In 2000, Taiwan enacted the first Hospice Palliative Care Act (HPCA) on self-determination of the terminally ill, which allowed palliative care to replace cardiopulmonary resuscitation (CPR) and other life-sustaining medical treatments during EoL. The individual is officially allowed to make an AD that is a "living will for hospice palliative care or do-not-resuscitate (DNR)" or consent for DNR signed by a surrogate, but this does not require participation in an ACP consultation [15]. A person who demonstrates legal mental capacity and is willing, may prewrite an HPCA AD under witness of two adults with full disposing capacity. The AD can be uploaded to the health insurance card system by a designated hospital or institution, without going through an ACP consultation. However, the HPCA only guarantees the right of medical decisions for terminally ill patients, which excludes patients with diseases that are incurable, painful, and difficult to judge as terminal.

We hope these revisions provide a clearer perspective of the difference between ACP, ADs as well as the difference between the HPCA and the PRAA, described on (Pages 5-6, lines 109-115):

PRAA Article 9 reads, “To establish an advance directive, the declarant must fulfill the following requirements”:

1. A medical institution has provided consultation on advance care planning to the declarant and affixed its seal on his or her advance decision.

2. The advance directive must be notarized by a notary public or witnessed by two or more persons with full disposing capacity.

3. The advance directive must be registered in his or her National Health Insurance IC card.

Comment 2c: *A CONSORT diagram is recommended to summarize the subject recruitment picture. “PWCIs” and “persons with dementia” were used interchangeably that may confuse the readers.*

**Response 2c**: The authors appreciate this suggestion. We have replaced the terminology for the family care receivers with persons with cognitive impairment (PWCI) throughout the manuscript. We have added a new Figure, which is a CONSORT diagram (Page 8). The figure is shown below.

**Figure 1**. CONSORT diagram for selection of dyads of family caregivers (FCGs) of persons with cognitive impairment (PWCI) participating in the ACP intervention.

Comment 3: *This study employed self-developed questionnaires as outcome measures. The items of each questionnaire were clearly presented, yet some of the contents and scoring method was unclear.*

Comment 3a: *For the 7-item scale for informal discussions about EOL care, the meaning of “informal discussion” was unclear. Please clarify if the ACP intervention itself a formal or informal discussion under your operational definition.*

**Response 3a:** Thank you for this suggestion. We have added “personal discussions” in the Abstract, Methods, Results, and Discussion. We have added section 2.4.4. entitled, “***Personal discussions about EoL-care***” (Page 12, lines 243-250) which reads as follows:

A 7-item scale for was developed to determine the number topics about EoL-care, unrelated to ACP that had been discussed between PWCIs and FCGs. Items completed the question, “I have had private discussions with my family member about. . .” 1) using a proxy signer, 2) DNR, 3) use of a feeding tube, 4) palliative care, 5) placement in a nursing home facility, 6) dispersal of money and personal belongings, 7) and funeral/burial arrangements. Items were scored either No (have not discussed = 0) or Yes (have discussed =1). Total scores ranged from 0–7; higher scores indicated a greater number of topics related to EoL-care that were discussed in private. The Cronbach’s alphas for PWCIs and FCGs were 0.88 and 0.85, respectively.

Comment 3b: *To measure the concordance on EOL care between the dyads, a hypothetical scenario was used. The ratings of the Likert scale from “0” to “5” were unclear. Would there be an option representing “not sure”? Such response was absolutely relevant and should be included. Please elaborate if the recoding method would have altered the original response.*

**Response 3b**: Thank you for this suggestion. We recoded the decision scores and revised the description (Page 12, lines 257 to 261):

The decision scores for each life-sustaining treatment were recoded and classified as either reject, accept or not sure. Therefore, a score = 0 was coded “1”, indicating rejection of the treatment; an item score of 1 to 4, was coded “2”, indicating not sure whether to accept or reject the life-sustaining treatment; and an item score = 5 was coded “3”, indicating acceptance of the life-sustaining treatment.

We have also revised the results in Table 6 (Please see Page 20).

Comment 4: **Result section**
Comment 4a: *Mind the way of presentation. Some of the results were not aligned with the sub-headings. For example, the t-test results of Familiarity of ACP should be put under 3.1.1, instead of 3.1.2. Same problem appeared in 3.2.1 from the FCGs’ perspective.*

**Response 4a**: Thank you for your suggestion. The t-tests for familiarity with ACP are now in section 3.1.1 (Pages 15, lines 300-303). Table 3 in on Page 16. Section 3.1.1 (Lines 300-303) reads:

We also compared pretest and posttest total scores and component scores for familiarity with ACP to determine if these changed post-intervention for PWCIs (Table 3). The total score for familiarity with ACP increased from 2.75 (SD = 3.40) to 5.31 (SD = 5.21) post-intervention (*p* < .001). Except for DNR, all component scores increased significantly (*p* = .01 to *p* < .001).

Section 3.2.1 has also been corrected for family caregivers (Page 18, lines 327-330):

When we compared total scores and component scores for familiarity with ACP pre- and post-intervention for FCGs (Table 5), the total score increased significantly post-intervention (t = -7.89, *p* < 0.001). In addition, all six component scores for increased significantly post-intervention compared with pre-intervention (*p* = .01 to *p* < .001).

Table 5 is on Pages 18-19.

Comment 4b: *Under 3.3, the interpretation of “consistency was significantly different between PWCIs and FCGs” were not supported by data.*

**Response 4b**: Thank you for noting the error in our description of the results. We have revised the scores in Table 6 (Page 20) as well as the paragraph (Page 20, lines 343-349):

Pre-intervention, only the consistency between PWCIs and FCGs for decisions about the use of CPR (65.9%) differed significantly (kappa *p*-value < .05). Post-intervention, percentages for consistency increased (ranging from 72.7-77.3%) as well as kappa values (ranging from 0.31-0.37). Although kappa p-values indicated significant increases post-intervention in consistency between PWCIs and FCGs for CPR, artificial respirator, and feeding tube between increased (all *p* < .05), the McNemar test indicated there was no significant difference in consistency between pre- and post-intervention scores (Table 6).

Comment 5: **Discussion section**
*Two references were used to compare with the results of this study on intention to engage in ACP. However, these two studies measured AD completion as outcome, which was far different from yours. Also, Lewis et al. (2015) reported that their recruitment period spanned through 8 months. The authors misinterpreted that their outcomes were measured at 8 months after the intervention. The conclusion on limited effectiveness for once-off ACP intervention to increase AD completion was not supported by the results.*

**Response 5**: Thank you for drawing attention to this misinterpretation. We have revised the paragraph (Page 25, lines 423-432)

Their results showed that 4% of FCGs and 9% of PWCIs completed AD documentation. Lo et al. (2017) provided 158 PWCIs with a psycho-educational intervention about ACP and 10.6% of participants chose to complete an AD after the 12-month intervention [31]. We speculate that the shorter 4-week follow-up period in our study may explain why only a few participants completed an AD post-intervention. Although recent studies suggest ADs are often triggered by a friend or family member experiencing health changes or a traumatic life event [33] and some individuals prefer informal plans over written AD documentation [30]. We did not collect information about changes in family members’ health status or preferences in AD documentation. Therefore, it will be important to include this information should be included in future ACP intervention studies.

Comment 6: *Regarding 4.3, “effect of the intervention on EOL care discussion,*

Comment 6a: *the topic sentence of this paragraph was not supported by results. Participation in EOL discussion for PWCIs did not show significant difference (p= 0.092).*
**Response 6a**: Thank you for your comments. We have revised the discussion section on the **Effect of the intervention on EoL-care discussions** (Pages 25-26, lines 443-446):

Only FCGs had a significant increase in participation in personal discussions about EoL-care post-intervention. These findings are in partial agreement with a meta-analysis of randomized controlled trials on the efficacy of ACP interventions, which demonstrated an increase in dialogue about EoL-Care decision-making between adults and family members [36].

Comment 6b: *Some discussions were made to explain the descriptive statistics of pre-test results, which was not aligned with the sub-heading.*

**Response 6b:** We have deleted discussion about the descriptive statistics of pre-test results and added the following (Page 26, lines 455-458):

Our ACP intervention included information about the symptoms of advanced dementia, such as eating problems and a decline in capacity for decision-making. This may explain the significant increase in post-intervention personal discussions for FCGs on the topics of feeding tubes and health care agents.

Pages 26-27, lines 468-470:

Although the post-intervention scores increased for the decision to reject life-sustaining EoL medical treatments and consistency for life-sustaining treatment decisions for both FCGs and PWCIs, they were not significantly different compared with pre-intervention scores.

Comment 7: *Overall, this paper provided very valuable data on the effectiveness of an ACP programme. The manuscript could be presented in a more logical way to enhance readers’ understanding. Also, the manuscript requires professional editing before it can be published.*

**Response 7**: Thank you again for your valuable comments. Our revised manuscript has been reviewed by a professional English-language editor.

**Responses to the comments from Reviewer 2 (Dr. Kenny)**

Comment 1: *First of all, the study was worthwhile to explore and provided a good intention to understand the advance care planning in Taiwan. Moreover, the data collection and results did support the findings to reveal particular situations of selected participants. Indeed, there are several points for the author's consideration and it may need for the author to improve for a better explanation of this topic.*

*The understanding between advance care planning and advance directive should be elaborated further. As the author focused much on medical perspectives and items in this research, it might need to let the readers have an explicit notification about the concept of advanced care planning, including social and medical aspects for a holistic care consideration. It might be good to clarify the ideas of advance care planning in the parts of discussion or limitation in this study;*
**Response 1**: Thank you for your comments. We have expanded the description of the difference between advance care planning and an advance directive in the introduction (Pages 3-4, lines 52-73).

Dementia is a progressive neurodegenerative disease that is life-limiting. An inevitable consequence of dementia is that the mental status of persons with cognitive impairment (PWCIs) will gradually deteriorate to the end-of-life (EoL) stage [1, 2]. Advanced dementia puts individuals at risk of inadequate EoL-care due to decreases in cognitive and decision-making abilities. Advance care planning (ACP) is one means of improving quality of EoL-care for PWCIs and their family caregivers (FCGs) [1, 3]. ACP is an umbrella term that includes personal, clinical, legal, and financial planning in preparation for the time an individual no longer has the capacity to make their own decisions about healthcare or legal proceedings. The process of ACP often includes advance directives (ADs), which are formal written documents describing preferences for future healthcare, including directives about do-not-resuscitate (DNR), intubation and artificial respiration, and tube feeding, as well as appointing a surrogate to make healthcare decisions in the event of incapacity [1, 2]. However, an individual can provide ADs without ACP, which is more extensive in terms of the number of personal preferences included.

ACP can be conducted formally through consultations with healthcare or legal professionals, or through informal discussions with family members [4]. A recent consensus paper defined ACP for health care as, ‘*the ability to enable individuals to define goals and preferences for future medical treatment and care, to discuss these goals and preferences with family and healthcare providers, and to record and review these preferences if appropriate’* [5]. National experts recommend that formal ACP should address the individual’s concerns and values across the physical, psychological, social, and spiritual domains and to tailor holistic care that responds to the wishes of the individual [5].

Comment 2: *One key element of this research was about "family-centered" approach and it will be necessary to explain this concept in this article. "Family-centered" intervention was one of the significant ideas to drive this study out and strongly advised the author to define or explain more in this aspect. The writing did not cover much or even limited discussion for this vital concept. The literature reviews or the discussion of findings were suggested to* *integrate understanding "family-centred" intervention. It would fulfil the reasonable expectation of readers and echo to author's topic with a particular "family centered" advance care planning intervention.*
**Response 2**: Thank you for your suggestions. We have added the significance of a "family-centered" intervention to the introduction (Pages 6-7, lines 130-139):

Studies have shown FCGs usually do not have adequate knowledge about the preferences of PWCIs for EOL-care [7, 20]. Therefore, when FCGs need to make EOL-care decision for PWCIs, they often feel uncertainty, stress, and guilt [21]. Family-centered care treats the family as a whole unit and embraces not only the view of PWCIs, but also family members [22]. Family-centered interventions may be more suitable for PWCIs because they can increase communication between the patient and family [23]. Family members, as well as FCGs acting as a health care agent, play an important role in helping PWCIs make future EOL-care decisions. Therefore, participation in the process of ACP can help FCGs better understand the values and preferences of PWCIs, which will be more conducive to the implementation of EOL-care decisions in accordance with the expectations of PWCIs [5].

We also have added a section to the discussion regarding the “family-centered” intervention (Pages 27-28, lines 490-501):

The design of the ACP intervention was guided by family-centered care, which included use of graphic manuals, simple text, and easy-to-understand terms to help both PWICs and their FCGs in understanding common life-sustaining medical treatments, and the procedures involved in formal ACP. The educational level of the PWCIs was lower than the FCGs, which could explain the lower scores for PWCIs compared with FCGs if they were less able to understand and absorb the contents of the intervention. PWICs were secure in expressing their personal thoughts and feelings regarding EoL-care due to the intimacy of their relationship with their FCGs, which lead to the acquisition of new knowledge between the dyads, despite the differences in dementia ratings. The family-centered intervention assisted PWCIs to participate in ACP and express their wishes about EoL-care to FCGs. Family-centered ACP should be conducted and promoted before the decision-making capacity of PWCIs is compromised, which will ensure that preferences are respected and fulfilled.

Comment 3: *The difference between people with mild cognitive impairment and people with dementia should make a warm reminder to the readers. As CDR 0.5 and CDR 1 included one key consideration of differences which was the status of "reversible" and "irreversible". A person with CDR 0.5 might have around 10-15% possibility of becoming dementia per year if there was no active intervention. The condition and context of this group of participants will not be similar to those who were at CDR 1 (diagnosis of dementia which was "irreversible"). Having a bit of clarification or even interest in exploring any difference between these two groups of participants. Their family caregivers might also have different interpretations and willingness of discussing advance care planning. As the study contained 34.1% (CDR=0.5) and 65.9% (CDR=1) of participants, the author might find out further in the results and findings of this research.*
**Response 3**: Thank you for your comments and suggestions. We compared pre- and post-intervention scores by group (section *3.4 Comparison the effect on PWCIs with different CDR scores):*

(Page 20, lines 353-358 and Table 7, page 21)

***3.4.1. Scale scores for PWCIs with CDR scores of 0.5 and 1.0***

We examined if the effect of the intervention differed between PWCIs with different scores on the CDR scale (Table 7). Pre-intervention, only familiarity with ACP differed significantly between PWCIs with a CDR = 0.5 and 1 (t = 2.12, *p* < .05). Post-intervention, there was no significant difference between the two groups of PWCIs any of the components of ACP (Table 7).

for FCGs (Page 21, lines 359-362 and Table 8, Page 22)

***3.4.2. Scale scores for FCGs of PWCIs with CDR scores of 0.5 and 1.0***

We also examined whether component scores differed between FCGs of PWCIs based on dementia ratings pre- and post-intervention. There was no significant difference for any variable between FCGs of PWCIs with a CDR score of 0.5 or 1.0 pretest or posttest (Table 8).

and for discussion (Pages 27-28, lines 485-498)

Earlier studies found milder impairment of cognitive function was associated with a greater likelihood of engagement in ACP [45, 46]. However, our study found the only difference between PWICs with a CDR score of 0.5 and 1.0 was for pre-intervention scores for familiarity with ACP. There were no significant differences for pre-intervention scores for FCGs of the two groups of PWCIs or post-intervention scores for PWCIs or FCGs.

The design of the ACP intervention was guided by family-centered care, which included use of graphic manuals, simple text, and easy-to-understand terms to help both PWICs and their FCGs in understanding common life-sustaining medical treatments, and the procedures involved in formal ACP. The educational level of the PWCIs was lower than the FCGs, which could explain the lower scores for PWCIs compared with FCGs if they were less able to understand and absorb the contents of the intervention. PWICs were secure in expressing their personal thoughts and feelings regarding EoL-care due to the intimacy of their relationship with their FCGs, which lead to the acquisition of new knowledge between the dyads, despite the differences in dementia ratings.

Comment 4: *For the consent form, the author might consider designing a unique consent form for people with dementia. In regard to the ethical consideration and a good standard of research involving people with dementia, a bigger size of wordings with simple terms and cue cards for people with dementia to understand the research might be offered. It might be an option for the author to think over in the future development of study relating to people with dementia;*

**Response 4**: Thank you for your reminder. Indeed, the IRB has specific requirements for the consent form for person with dementia in Taiwan, which includes wording that is easy-to-read. Thus, we added this information to the procedures (Page 9, lines 177-179)

The IRBs in Taiwan require consent forms involving PWCIs that are easy-to-read, which requires a large sized font and wording that uses simple easy-to-understand wording to describe the purpose and procedures of the study.

Comment 5: *For the discussion part, the author mentioned the consistency between PWCIs and FCGs for life-sustaining treatment decisions. It would be great if more reviews could be included for understanding this discrepancy. The educational levels of the participants in this research could be considered and how "family centered" interventions could improve this situation would be valuable discussion in this manuscript. The author identified this remarkable area to explore and it would be excellent if the author could provide some of the reflections as well as recommendations in this part.*
**Response 5**: Thank you for your suggestions. We have added a discussion of our family-centered intervention (Please see **Response 2**, above). We also added comments about the educational levels of the participants in the discussion (Page 28, lines 493-495):

The educational level of the PWCIs was lower than the FCGs, which could explain the lower scores for PWCIs compared with FCGs if they were less able to understand and absorb the contents of the intervention.

In the limitations, we added the following

Page 28, lines 503-507:

First, a single-group paired with pretest-posttest design was used and data were collected from participants recruited from only regional hospitals in northern Taiwan and this Metro Area of Taipei has a population with high levels of education. Thus, our findings might not be relative to other areas or with lower levels of education.

And Pages 28-29, lines 514-517:

Fourth, hypothetical life-sustaining treatment decisions and ACP intentions may not match real-life situations or the educational level of participants. We recommend the model of the ACP intervention and the material provided be further tested to determine whether these are suitable for people with different backgrounds and levels of education.

Comment 6: *Minor typo at row 430.*
**Response 6**: Thank you for noting our error. We have corrected the typo. Please see Page 27, line 477.

Comment 7: *For the limitation part, it would be good to report 16 FCGs who refused to participate in this study. If possible, the questionnaires could add some questions to understand the reasons which might help the author explore further in this topic. It will be fascinating to explore the reason for withdrawal in each stage to benefit the future design and relevant research findings. It also contributes to the accumulation of knowledge.*
**Response 7**: Thank you for your suggestion. We have revised the limitations (Page 28, lines 510-514):

The issues of EoL and death remain culturally sensitive topics in Taiwan and reluctance to discuss EoL might have been the reason 16 FCGs refused to participate. Therefore, we suggest adding questions about why FCGs refused participation or withdrew at each stage, which could benefit the future design and relevance of the research findings.

Comment 8: *The author might consider suggesting the optimal time to discuss advance care planning in dementia care, i.e. during the post-diagnostic period or whenever the health condition was changed, etc. The conclusion might be supported by previous literature for echoing the critical ideas of "family centered" interventions in this study.*

*Thanks for conducting this study which contributed to the development of advance care planning for people with cognitive impairments. That would be a significant area to explore more understand and shared practical wisdom in the coming time. Good luck and best wishes~~*

**Response 8**: Thank you very much for your suggestion. We have added the optimal time to discuss advance care planning in dementia care to the discussion (Page 25, lines 436-438):

Past studies suggest that ACP in dementia care should be started as soon as the diagnosis is made and then revisited with both PWCIs and their family members on a regular basis and following any significant change in health condition [1, 23].
